# Use of an Internet “Viral” Marketing Software Platform in Health Promotion

**DOI:** 10.2196/jmir.1127

**Published:** 2008-11-26

**Authors:** Pierre Gosselin, Philippe Poitras

**Affiliations:** ^2^WHO Collaborating Center on Environmental and Occupational Health Impact Assessment and SurveillanceCentre hospitalier universitaire de Québec (CHUQ)Québec CityQuébecCanada; ^1^Institut national de santé publique du QuébecQuébec CityQuébecCanada

**Keywords:** Internet, viral marketing, email marketing, email, health promotion, person-to-person communication, viral campaign

## Abstract

**Background:**

Health-related websites have become a common tool for public health authorities to inform the general public of their health promotion information and programs. However, building traffic in the cluttered health Internet universe is becoming increasingly complex, costly, and challenging for governmental health promotion websites. In 2006, the Canadian Health Network (CHN), a cooperative program made up of the Public Health Agency of Canada (PHAC) and some 20 health non-governmental organizations (NGOs), was looking for an affordable marketing tool for the promotion of its website and contents to specific populations.

**Objective:**

To test new and innovative marketing tools for a health promotion website in Canada.

**Methods:**

Within the context and constraints of a governmental health promotion website, an adaptation of a commercial Internet viral marketing software platform was developed and implemented under the name “The Crazy Race”. This process was done interactively between seven NGOs and the CHN staff. The communication objectives were (a) to provide a meaningful visit that could communicate important public health messages, and (b) to increase subscriptions to its e-newsletter. A nine-step standardized Web-user experience (Internet path) was thus defined and experimented with under a pre-determined operating budget of less then Can$50,000, mainly paid for by participating organizations on a pay-per-performance basis.

**Results:**

An initial group of 215 people were sent an invitation to participate in the campaign. Over its 15-day duration, the campaign generated by itself and without any media support a total of 110,200 Web user participants who registered and sent a total of 439,275 invitations (2% of the Canadian Web-user population of 21.8 million in 2006). The epidemic self-dissemination of the campaign occurred in both French and English populations and spread across all age groups. Two-thirds of the participants were women.

**Conclusions:**

The use of an Internet viral marketing platform proved to be effective in bringing thousands of Web users to discover and explore a governmental health promotion website. The exponential growth of the person-to-person dissemination generated by the campaign indicates that public health messages have high viral propagation potential on the Internet (“virulence”) when they are presented in the context of an enjoyable online game. This could constitute a promising method to create affordable mass audience public health campaigns, both in Canada and internationally.

## Introduction

Offering and maintaining consumer health websites has become a practical and cost-efficient mechanism for public health agencies to communicate health promotion information and programs to the general public. The Internet allows content to be updated instantly and inexpensively in comparison to the available alternatives (eg, paper brochures, CD-ROMs, and software applications, etc) [1]. The Internet has been adopted worldwide over the past decade, including 7.9 million Canadian households in 2005 [2], for a total online population of 21.8 million Web users in 2006, of which 87% had a broadband connection [3]. Furthermore, 58% of Canadian Web users searched for medical or health-related information at home in 2005 [2], indicating a strong demand for online consumer health information and resources.

However, to build traffic in the current Internet universe is a complex and costly challenge for any consumer website, especially in the cluttered health sector. Web users currently have access to more than 25,000 health-related websites in North America alone [4], ranging from general governmental consumer health websites (provincial ministries and state or federal agencies), to websites on diseases, specific topics/groups, and non-profit websites (eg, associations dealing with diabetes, cancer, asthma, mental health, children, women, and seniors, etc) to websites for pharmaceuticals and pharmacists’ websites. Health communicators face three challenges when marketing a non-revenue generating consumer health website in order to increase or maintain traffic and to build recognition (branding).

Firstly, the costs of media advertising for radio, TV, newspapers, Internet banners, pay-per-click search engines, and/or email marketing to promote and market a consumer health website are now very high, if not prohibitive, for any governmental public health agency. This is especially true in the context of a geographically widespread country such as Canada, where marketing operations have to be executed in a multilingual and multicultural context and where different segments of the population are targeted (women, men, youth, seniors, Aboriginal peoples, and people living with disabilities, etc). For instance, in 2006 such costs were estimated on average to be $60 per new customer using email in the United States, $8.50 when using search engine strategies such as Google Ads, and approximately $50 for online display ads such as banners [5,6,7].

Secondly, Internet users visit websites they are used to and those offering the contents they seek. When users are searching for new websites they usually do so by typing a keyword or topic in a search engine (proactive action), clicking on a banner (spontaneous action), or requesting a friend’s advice via electronic communication, face-to-face communication, or community websites (word of mouth). However, the latter would not likely happen often nor spontaneously for a consumer health website, since health brands are one of the less discussed between consumers, along with other private matters such as finance, home, household products, children, and lifestyle [8].

Thirdly, Web users do not pass along information within their social network if this information does not convey strong viral dissemination potential, for instance messages that are not intriguing, funny, or utilitarian in nature [9,10]. Therefore, public health or health promotion information, which is both serious and private in nature, possibly falls within this low-potential category for being passed along. This becomes a real problem in the current cluttered Internet universe where individuals tend not to pass along messages or even consider email messages that are not received from someone they know and trust.

The field of viral marketing based on person-to-person electronic communications is being increasingly explored and experimented with in the commercial arena to promote and market products or service-related websites. The terminology “person-to-person” or “peer-to-peer” used throughout this paper strictly refers to the phenomenon of people sending email invitations to other people in their close personal social network to play an interactive online game with them on the topic of health, and does not refer to other peer interactions, be they online or offline [11,12]. These viral marketing approaches, tactics, or applications are deployed with the aim of disseminating messages within whole populations or targeted segments of a population with the ultimate goal of gaining new customers. However, success in spreading out across populations varies greatly and is impossible to predict a priori for any given attempt. As mentioned by Phelps et al, “viral marketing remains a relatively neglected academic research topic. In addition, it is recognized as being one of the most inexpensive and efficient methods for organizations to differentiate themselves and to reach their clientele” [9,13].

This paper describes a viral marketing experiment performed in February 2007 to promote a free, Canadian federal government consumer health promotion website offered to English- and French-Canadian populations. This project consisted of transposing and adapting an Internet viral marketing software solution to a public health context, one initially engineered to market merchants, products, or services in the commercial arena. The experiment launched a consumer health promotion website with two of its key topics and functions being to gain new, regular Web users through new subscriptions to the e-newsletter and to communicate important health promotion or public health messages to campaign participants by enticing them to complete short quizzes and win “healthy” prizes.

## Methods

### Context and Constraints of the Promoter

To help improve the health of individuals, the Canadian Health Network (CHN) website had to become known by the largest number of people possible in order to maximize its impact. The CHN was a consumer health promotion website operated from 2001 to 2008 by the Public Health Agency of Canada (PHAC) which has since merged with other PHAC websites. The CHN’s mission was to promote healthy choices by communicating trustworthy information on health promotion, disease, and injury prevention through a network of specialized, expert non-governmental organizations (NGOs). It provided free access to more than 20,000 English and French peer-reviewed, Web-based resources on 25 key thematic health topics and population groups. This formal peer-review process was a key, distinctive feature of CHN providing quality assurance, credibility, currency, and relevance of the content.

To carry out its mission and increase the number of its users, the CHN faced three key marketing challenges, within the constraint of a modest budget of less than Can$50,000. The first of these challenges was to reach out to as many Web users (consumers) as possible across Canada in both English and French; secondly, to attract consumers to its website for a meaningful visit to exhibit its main features; and thirdly, to convince them to return by demonstrating its relevance and to build loyalty by having them subscribe to its bi-monthly e-newsletter.

An experiment was thus undertaken which consisted of using a spin-off application of a patented Internet viral marketing software platform powered by a Canadian company named YOUge.com Inc. [14,15]. Previous applications of this platform had been successfully applied in the commercial arena to promote product or services websites. Adapted for the CHN, the viral marketing platform adopted the form of an online game using the metaphor of a positive and healthy activity (ie, a real-time virtual human foot race between friends).

### The Objectives of the Experiment

Within the framework of a pay-per-performance and pre-determined budget, the objectives of the experiment were set as follows:

- Facilitate the CHN’s ability to attract as many Web users as possible to its website for a meaningful first-time visit during which its key features can be exhibited.
- Communicate public health messages to Web users in the following seven areas through the completion of short health quizzes: active living, cardiovascular health, environmental health, respiratory health, healthy eating, injury prevention, and HIV/AIDS.
- Increase significantly and at a sustainable rate the number of subscriptions to the CHN e-newsletter.
- Increase the general level of awareness and recognition of the CHN within English- and French-Canadian populations.

### The Internet Viral Marketing Software Platform

The experimentation process consisted in the following nine phases, as follows.

The first phase consisted of adapting and transposing the viral marketing Internet software platform, based on sophisticated and patented mathematical algorithms [15]. The system used patented algorithms allowing participants in the race to overtake the person who invited them and win the race by attributing the value of a participant’s individual electronic word of mouth to the one who actually generated it. This unique feature made the game fun and exciting because anyone could win their race, depending on the number of active participants they generated directly (via invitations to their friends) and indirectly (via invitations sent by their friends, by friends of their friends, and so on). More specifically, these algorithms fostered the following behaviors or benefits. Firstly, they enticed game participants to select and invite competitive people. The more motivated competitors that participants had in their race, the faster their runners ran, and the greater were their chances of winning the race. Secondly, the algorithms made it counter-productive for participants to register more than once for the game. Thirdly, for the campaign sponsor (CHN), the algorithms ensured that the number of prizes awarded to winners was exactly correlated to the number of participants. This enabled budget control based on a pay-per-performance formula.

**Figure 1:**
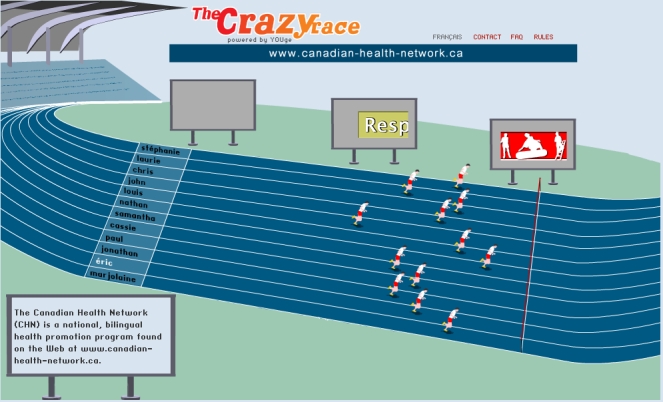
Online, real-time interface for foot race between friends

**Figure 2:**
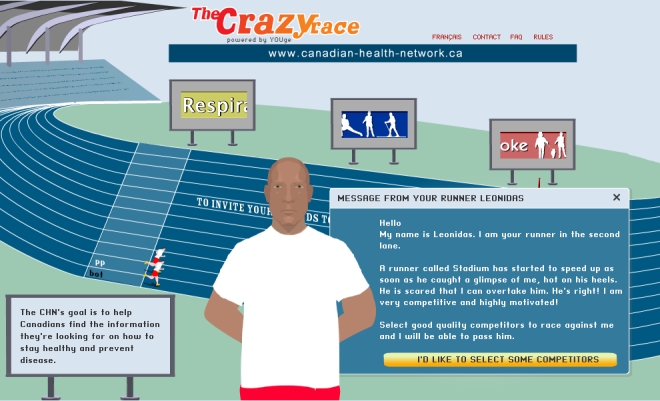
Friendly virtual runner named Leonidas

**Figure 3:**
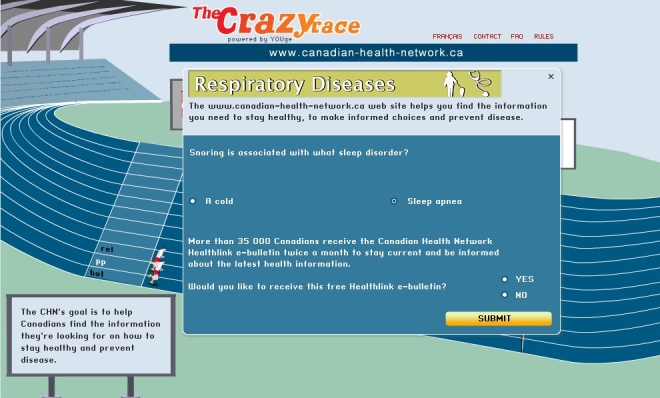
A health quiz on respiratory health

The second phase consisted of designing a Macromedia Flash graphical interface with the following features. The first one was that of a game personifying a real-time foot race between friends encouraging person-to-person pass-along behavior via email invitations (Figure 1). The second feature needed to be as user-friendly as possible to maximize overall participation levels. This was accomplished through the design and programming of a friendly virtual runner named Leonidas who ran on behalf of participants and guided them, via a one-to-one dialogue box, through different stages of the race. Leonidas had three key functions: (a) entice participants to invite good competitors; (b) encourage participants to improve their health by completing up to three health promotion quizzes (embedded incentives) (Figure 3); and (c) guide participants in choosing the best course of action at any given moment during the game (Figure 2).

The third phase included developing text (in both English and French) in order to entice participation and reinforce the importance of taking care of one’s health. This included text for game website pages and all email messages, including invitations to join the game, post-registration confirmation, follow-up communications, and reminders.

The fourth phase included designing and programming an intelligent architecture and database system to support the following features: Leonidas’ key roles and functions; real-time updating of the races; the invitation panel (engine); real-time online chatting between participants; and the equal distribution of up to three health promotion quizzes per participant among the seven quizzes developed for the campaign.

In phase number five, a prize reinforcing health promotion was prepared and offered to the campaign’s first 500 winners. The health prize consisted of a reusable water bottle, a shoe bag, and a book of healthy recipes (approximate value of Can$25).

The sixth phase centered on coordinating the involvement and input of the seven public health-expert NGOs involved in the project from across Canada, along with the Public Health Agency of Canada, namely: the Canadian Public Health Association (HIV/AIDS); CHUQ WHO Collaborating Center (environmental health); Dietitians of Canada (healthy eating); SMARTRISK (injury prevention); the Canadian Lung Association (respiratory health); Alberta Centre for Active Living (active living); and Capital Health Edmonton (cardiovascular health).

Phase seven consisted of defining the topics, format, literacy level, and copywriting of the seven health quizzes to be delivered to participants, promoting timely and easy-to-remember health promotion messages.

Phase eight launched the campaign under the name “The Crazy Race” in English and“La course folle” in French on February 8, 2007, by sending 215 email appeals to people involved in the eight partner organizations and the CHN.

Finally, phase number nine consisted of overseeing the campaign’s evolution and analyzing its results and performance. The campaign was ended on February 23, 2007 with the occurrence of the 500th winner.

### The Web Users’ Experience

The campaign was disseminated by thousands of people who joined a friend’s race after receiving and accepting an email invitation, and who did the same by inviting friends by email to join their race. Each participant’s experience followed a nine-step standardized path, as described below.

In step one, Web users receive an invitation by email from a person they know with the name of that person appearing in the “From” field, thus confirming that the invitation is not spam. The invitation is personalized with text calling for potential participants to join the race of their friend.

In step two, Web users click on the embedded hyperlink appearing in the invitation message.

In step three, Web users land on the campaign website’s homepage where they are invited to register and play.

In step four, while registering, they type in their name, gender, age group, postal code, and email address. After clicking on the registration button, the system sends them a personalized confirmation email with a link to access their race directly at anytime, and the Web users become registered participants in the campaign. Once participants are registered with a given email address, the system blocks any further invitation sent by friends to this email address by indicating to them that the participant is already registered. Also, the system does not allow a second registration with any email address which is already registered.

In step five, new participants are shown a racing track which contains the first name of the friend who invited them in the bottom lane and their own name in the second lane. The virtual runner Leonidas appears on the screen and introduces himself. He explains that in order to accelerate and win the race, they need to run against competitive runners, and he asks participants to invite the best competitors from amongst their friends. Upon reading the rules, participants will intuitively understand that it is counter-productive to register more than once and that the payoff comes from inviting other runners.

For step six, an invitation panel is presented from which up to ten people (friends, relatives, and colleagues, etc) who may like to be involved can be invited. Participants type in the first name and email address of each guest, choose the appropriate language (French or English), and click on the “Send” button. The invitations are sent by the system and the recipients begin at step 1. Once invitations are sent, participants will observe that Leonidas has progressed forward in his lane. (Each time invitees register with the game, the system automatically sends an email from Leonidas to participants to inform them that a new competitor has joined their race. The more competitors who join the race (up to a maximum of ten) for any participant, the faster Leonidas runs for them. This is the key function of the algorithms described above.

For step seven, after participants have invited at least one potential competitor, Leonidas explains that staying healthy is needed to accelerate in the race. He adds that one of the CHN affiliates (the seven organizations mentioned above) wishes to take care of their health and well-being. He then prompts participants to click on a quiz banner to complete a short health quiz that could improve their health and help them reach their full speed potential.

In step eight, a quiz panel pops up and presents a question with a hint provided as to the location of the answer on a CHN Web page followed by a “Find the answer here” button. By clicking on this button, participants are automatically sent to one of the CHN’s topic homepages (one of the seven topics mentioned above) where the answer can easily be found. This provides a unique visitor count on the CHN website for the first quiz. With the answer in mind, participants close the CHN page, return to the race website, and are offered a choice of two answers where they check one, thus providing a correct or incorrect response. If incorrect, the system tells the player and sends the player back to the related CHN webpage so that the right answer will eventually be checked. If answered correctly, participants will observe that Leonidas has moved forward in his lane, realizing the immediate impact of completing the health quiz. This process provides a positive action-reaction pattern and a strong indication that the health message may have been learned, although this cannot be measured in the race. In addition, participants are asked if they would like to subscribe to the free CHN e-newsletter. This appeal is timely, being offered immediately after participants spend a few minutes exploring the health website to locate the quiz answer. If “Yes” is checked, this is counted as an opt-in (a high value, permission-based email address for CHN). If “No” is checked, they are asked again after completing the second and third quizzes, but with a different question (ie, a new trigger) each time.

Finally, in step nine, each time participants verify the status of their race or accomplish an action, Leonidas provides them with timely, tailored messages. The objective is to have participants complete up to three health quizzes and invite up to ten competitors. Ultimately, as more Web users were invited, registered, invited other competitors, and completed quizzes, the campaign process generated thousands of parallel races unfolding simultaneously. Considered all together, these races were evidence of the naturally growing viral dissemination of the campaign throughout the population.

## Results

### Epidemic Self-dissemination

**Figure 4:**
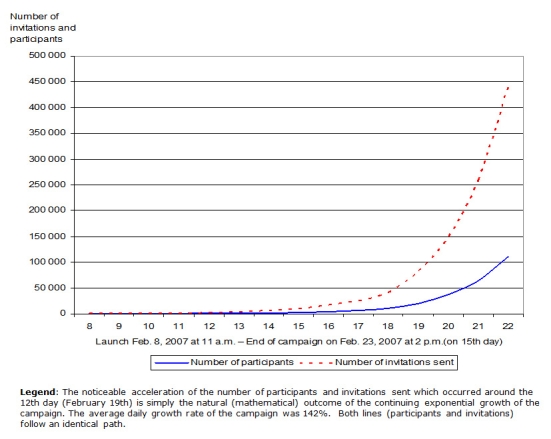
Exponential growth of registered participants and invitations sent

In only 15 days, the campaign generated, without any other offline or online media support than the invitation engine used by game participants, the following results. After the initial 215 invitations were sent to staff of participating CHN affiliates, a total of 68 people were registered and 679 invitations were sent on the very first day of the campaign. After 15 days, a total of 110,200 Web user participants were registered and a total of 439,275 invitations sent through their personal and private networks (Figure 4). Although it was impossible to assess precisely whether each registered participant corresponded to a unique individual, the system characteristics described above certainly acted to keep the number of duplicate registrations very low. The overall daily growth rate of the campaign was an exponential 142%, thus producing an epidemic dissemination within the Canadian population. In other words, the campaign grew naturally by itself and was terminated before reaching saturation in order to respect the fixed budget. The epidemic dissemination occurred in both the French and English populations.

Participants were fairly evenly spread across all age groups (ie, 11% in the 13-17 age group, 30% in the 18-24, 29% in the 25-34, 12% in the 35-44, 11% in the 45-54, and 7% were over 55). Women represented 66% of the campaign participants.

### Key Performance Indicators Compared to Initial Objectives

**Figure 5:**
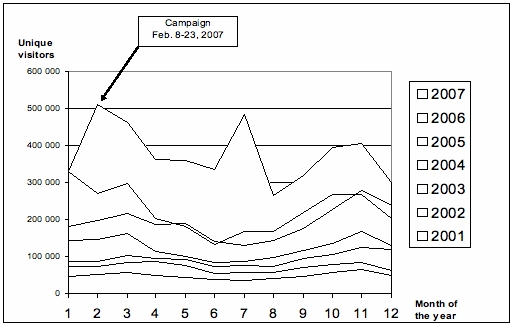
Unique visitors’ annual growth from 2001 to 2007

The first objective was to draw as many Web users as possible to the CHN for a meaningful initial visit to show them the website’s features and relevance. Approximately 300,000 visits occurred on the CHN website during the campaign, mostly made by new, unique visitors generated by the game who came to the site to find answers to their health quizzes (Figure 5). The CHN defined a visit as a series of actions that begins when a visitor views any first page from the server and ends when the visitor leaves the site or remains idle beyond the default 30 minute idle-time limit. Unique visitors were defined as individuals who visited the site during any (monthly) reporting period and were only counted the first time they visited by noting their IP address.

**Figure 6:**
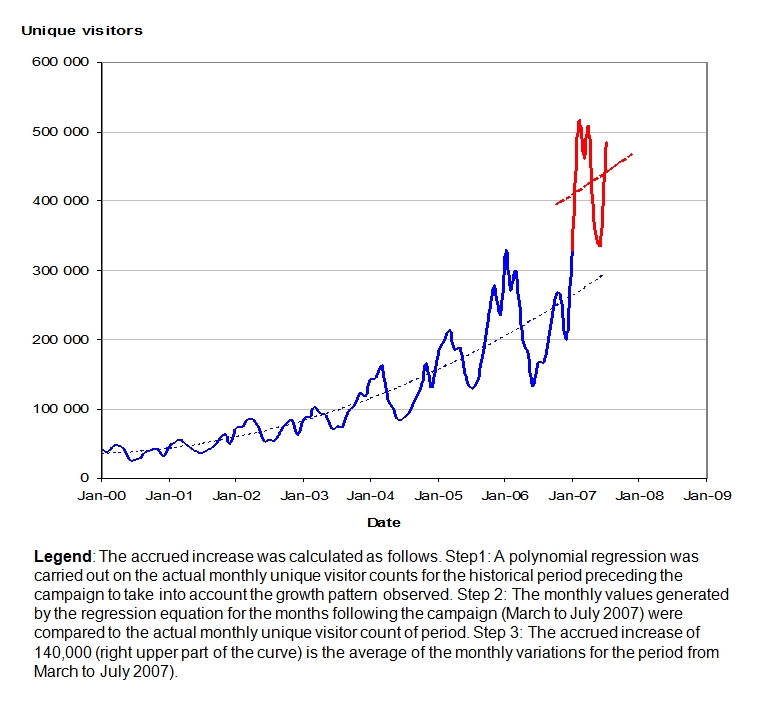
A post campaign accrued increase of unique visitors per month was observed

In addition, an estimated average accrued increase of 140,000 unique visitors per month was observed after the completion of the campaign during the period from March to July 2007 (Figure 6). This accrued increase means that the growth in traffic exceeded what would have been expected by applying the average traffic growth rate trend of that time. Noticeably, this occurred without any other unusual marketing or promotion initiative from the CHN.

The second objective was to increase significantly, and in a sustainable way, the number of subscriptions to the bi-monthly CHN e-newsletter. A total of 31,866 participants registered to the e-newsletter, doubling in two weeks the total number of subscribers reached in the previous six years. The conversion rate was 45%, meaning that 45% of the participants who had completed at least one health quiz and were subsequently asked to subscribe chose to do so (gave permission to be sent the e-newsletter). Only 2% of these new subscribers unsubscribed after nine months. Although this does not necessarily mean that the 98% who remained subscribed actively read or paid attention to the bi-monthly e-newsletter, we believe that these new subscribers are probably behind the sharp and sustained increase in the website traffic mentioned above.

The third objective was to communicate health promotion messages through the completion of health quizzes. A total of 70,580 people (64% of the participants) successfully completed between one and three health quizzes. On average, each quizzer completed 1.92 quizzes. In total, 135,395 health quizzes were successfully completed during the 15-day campaign.

The fourth and less tangible objective was to heighten the level of recognition of the CHN website within the Canadian population. The magnitude of the results described above establishes the likelihood that the campaign managed to increase the recognition of the CHN website within the Canadian population. Indeed, more than 400,000 people saw or interacted with the CHN brand, either through the invitation they received from a friend or more actively by participating in the campaign. This corresponds to 2% of the Canadian Web-user population of 21.8 million at the time of the experiment [12]. In addition, two questions posed to those who subscribed to the CHN e-newsletter reinforced this assertion and indicated that the campaign fostered loyalty to the CHN. To question 1, “Had you visited the Canadian Health Network before playing this game?”, 63% of the 18,480 people who were asked this question responded, “No”. To question 2, “Will you visit the Canadian Health Network (CHN) again in the future?”, 92% of the 13,085 respondents said, “Yes”. More importantly, 89% of those who responded “No” to question 1 answered “Yes”, that they would visit the CHN again. This tends to confirm that the campaign provided valuable exposure of CHN benefits for game participants, thus leading to a positive perception and high appreciation of the CHN.

## Discussion

### Internet Marketing in Health Promotion

The use of an Internet viral marketing software platform generated outstanding marketing outcomes for the CHN website. These results are interesting both in terms of the epidemic dissemination of health promotion related messages and in terms of the efficacy and efficiency demonstrated in acquiring new and returning Web users. Results may be considered particularly positive for a governmental health promotion website working with a small marketing budget. This experiment represents a first in Web-based, person-to-person viral marketing health promotion campaigns.

In 2006, Suggs presented a 10-year review on the body of research available on the use of new technologies in health communication, including computer-based approaches [1]. The review focused on studies that reported positive outcomes. The review provided insights as to how new technologies were used to communicate health messages and what their outcomes were in terms of supporting or improving health communication efforts. Among the findings, it was noted that the use of new technologies was growing in virtually all areas of health communication, including consumer and health promotion. It also identified computer technology as the most commonly implemented new technology in the past decade and concluded that “most of the innovation in technology-based health communication has been computer driven*”* [1]. However, the applications included in the review were limited to websites or portals, Web technologies, or Web communications supporting patient-provider interaction or designed to provide education and social support to influence behaviors or increase self-efficacy, with no mention of viral or person-to-person pass-along applications.

Despite the fact that there was no mention of any person-to-person (or peer-to peer) email communication applications, Suggs concluded “that the past decade of research in health communication demonstrates that technology has been used successfully to deliver a variety of messages using multiple mediums” and that further research should strive to answer research questions such as how better to tailor communication content or what technology “channels are most effective in communicating the health message for what populations and for which health topics” [1]*.*

This suggests that the CHN’s person-to-person email pass-along experiment used to raise the profile of its consumer health promotion website and communicate public health messages could have been the first project of its kind to have been carried out in the health communication arena which utilized measured self-dissemination results to propagate public health messages. To the best of our knowledge, it is also likely that the experiment identified a new and singular Internet-based channel (ie, a person-to-person pass-along email channel of communication through an Internet viral marketing game platform) to communicate efficiently health messages both in terms of reach and costs, as compared to the available alternatives.

Another interesting angle to examine the singularity of the experiment is to compare its dissemination patterns with what can be found in the existing literature regarding person-to-person electronic communication. According to Léger and Scholz, North American consumers are bombarded with approximately 4000 publicity and promotional stimuli everyday, including electronic ones [16]. Organizations are thus looking for innovative and effective approaches to reach their clientele. Viral marketing through email and other means is one of the approaches that is being explored by marketers from all sectors. Viral marketing is a “phenomenon that facilitates and encourages people to pass along a marketing message voluntarily” [17]. More specifically, the “term refers to marketing techniques that use pre-existing social networks to produce increases in brand awareness, through self-replicating viral processes, analogous to the spread of pathological or computer viruses” [18].

According to Phelps et al [9], marketers use viral marketing to increase product or service knowledge and awareness. Despite the fact that it has attracted a great deal of attention in the marketing industry, viral marketing remains a relatively neglected research topic and almost nothing “is known about the motivations, attitudes, and behaviors of the people (those sending the email to others) that constitute the essential component of any such strategy”[9].

Among the factors explaining a Web user’s inclination to circulate information, a range of motivations such as the desire to help, a financial incentive, or having a sense of providing a social benefit to the community have been mentioned [19]. Others have also provided insightful observations on the behavior which motivates people to pass along information by email or other, consumer-mediated, consumer-to-consumer tools, as well as observations on some characteristics of the most passed along types of email messages and other conditions inclining people to forward email messages [9,10]:

- Consumers are much more reluctant to delete a message received from a person they know. Moreover, when a message comes from a friend, there is an implicit level of credibility attached to it, and people assume that the information is of value.
- Email messages received from close, interpersonal sources have a greater chance of being forwarded than messages from unfamiliar impersonal or commercial sources.
- People experience positive emotions when they send pass-along emails. They might feel excited, helpful, happy, or satisfied. The five most reported motives for passing along an email are because it’s fun, enjoyable, or entertaining; because it may help others; and because it promotes having a good time.
- Women are more likely than men to pass along email messages.
- People using an Internet broadband connection are more willing to forward messages than those accessing Internet through dial-up modems.
- Messages of a utilitarian or hedonic nature, or that spark strong emotions such as humour, fear, sadness, or inspiration are more likely to be forwarded.
- Consumers are irritated with unsolicited emails received from companies or organizations, and they usually delete these without opening them. Thus, “people are not likely to forward emails from companies because they consider the information company-produced ‘junk’” [9].

Finally, Phelps et al, in their exploratory work, found that jokes were the type of content being forwarded most often, making up close to 50% of this content, and games-related content represented only 1% of emails received by a sample of recipients [9]. They also suggest that offering compensation or incentives to entice consumers to pass along email messages could dilute the power of the recommendation if the recipients were aware of it.

Before comparing the above literature with the pass-along email patterns of the Internet viral platform used by the CHN, it is important to consider how it differs from pass-along email per se. Firstly, the participants had to register with the campaign before being able to invite their friends through the software platform. This represents an extra step as compared to simply forwarding emails from a computer email manager. Secondly, the invitations were designed to encourage participation in a popular online foot race between friends, rather than to promote health or the CHN directly, although the latter’s brand was mentioned in the invitation copy. It is the game and the fun associated with it that caused the viral message to be passed from person to person in a participant’s social network. The introduction to the CHN (ie, the advertisement) was embedded in the game platform and experience. Finally, with each invitation to friends to join their race, campaign participants generated a de facto epidemic dissemination of the game message, which in turn attracted more than 100,000 registered participants to the game. By playing the game, they then discovered the CHN’s main features and eventually became new consumers of the CHN website.

Interestingly, the dissemination patterns generated by the use of an Internet viral game software platform used to promote the CHN appear to coincide with the above literature. First of all, participants of the online virtual foot race came to the game by linking from what would have been perceived to be a utilitarian or hedonic email invitation received from an interpersonal source and not from an organization trying to promote itself. This meets most of the conditions presented above for the most forwarded types of email messages.

Secondly, the point of the invitation was to invite friends to play a healthy online game which would be useful to anyone’s health. This game environment likely provided senders with both the opportunity to have fun with their close social network in an enjoyable setting and an occasion to help others. In other words, the online game setting clearly appealed to participants’ desires for fun, entertainment, and social connections which are the foundation of consumers' motivations regarding pass-along email. Moreover, 66% of the game participants were women, thus corroborating previous observations that women are more likely to pass along messages [9]. Also, the campaign took place in Canada, where 87% of online households use a broadband connection [13] which is a factor known to facilitate pass-along email.

Finally, the observed results of the CHN viral experiment seem to contradict the suggestion that offering compensation or incentives with pass-along emails may negatively impact the pass-along potential of a communication. On the contrary, the self-epidemic dissemination of the experiment, which included both the possibility of winning a healthy gift package by competing with friends and answering health quizzes, and the use of mathematical algorithms which subtly provided incentives to pass along email, clearly demonstrated that these factors were, at the very least, not an impediment to ”virality”.

### Limitations and Further Research

The experiment with the Internet viral marketing platform proved to be effective in actually propagating useful public health messages within the Canadian population and in significantly increasing the CHN’s website traffic. However, its impact on behavioral change for CHN’s new Web users, and its a posteriori potential health improvement effect, could not be measured nor assessed either quantitatively or qualitatively. In this regard, this peer-to-peer emailing experiment does not add to the scarcity of knowledge and understanding of the factors and conditions influencing health outcomes from peer education, peer-to-peer interactions, or virtual communities in health as described by Milburn [12] and Eysenbach [11].

The marketing results of the experiment highlighted in this paper should not be used to predict results for any further use of the same platform for other health promotion websites. Indeed, the experiment was completed at a specific time and in a specific media environment with specific topics, and the campaign self-disseminated within specific interpersonal social networks, reaching specific individuals in specific age groups. In fact, the use by the CHN of the same Internet game platform in 2008, with only slight modifications to the scenarios and scripts, delivered a completely different pattern of epidemic self-dissemination, and in different age groups, but with very similar overall marketing outcomes.

Although the use of the Internet viral marketing platform proved to be a promising breakthrough for the efficient marketing and branding of a governmental health promotion website, further experiments and research are necessary. This work will help to assess more thoroughly the extent of the utility, efficacy, and limits of the tool to drive traffic and build loyalty for health promotion websites; to deepen our understanding of the gaps observed in dissemination rates between age groups and languages (French and English populations); and to understand better the Web user’s behavior while using the game platform, as well as the conditions, factors, and drivers behind the epidemic self-dissemination of the game within personal social networks.

## Abbreviations

CD-ROM: compact disc read-only memory
CHN: Canadian Health Network
CHUQ: Centre hospitalier universitaire de Québec
NGO: non-governmental organization
PHAC: Public Health Agency of Canada
WHO: World Health Organization
